# Case Report: Delayed complete transection of the sigmoid colon after severe pelvic fracture leading to septic shock

**DOI:** 10.3389/fsurg.2026.1984207

**Published:** 2026-10-07

**Authors:** Yunlong Liu, Mufan Li, Cheng Sun, Min Zou

**Affiliations:** 1 Clinical Medical College, North Sichuan Medical College, Nanchong, China; 2 Department of Orthopedics, Sichuan University Affiliated Chengdu Second People’s Hospital, Chengdu Second People’s Hospital, West China School of Medicine, Sichuan University, Chengdu, China

**Keywords:** delayed complete transection of the sigmoid colon, multidisciplinary collaboration, multiple organ dysfunction syndrome, septic shock, severe pelvic fracture

## Abstract

Severe pelvic fractures caused by high-energy trauma may be complicated by bowel injuries. These injuries may be evident at initial presentation or remain occult, resulting in delayed diagnosis. Delayed complete transection of the sigmoid colon associated with pelvic fractures is extremely rare. We describe a 57-year-old woman who was admitted to the Intensive Care Unit (ICU) following a traffic accident that resulted in a Young–Burgess lateral compression type III pelvic ring injury, hemorrhagic shock, impaired consciousness, and multiple associated injuries. Abdominal CT on post-injury days 1, 2, and 3, emergency focused assessment with sonography for trauma (FAST), and repeated bedside ultrasonography showed no definite bowel injury. Bowel sounds were normal on admission, reduced on day 2, and absent from day 3, but abdominal symptoms could not be assessed reliably. Some inflammatory and organ-function indices partially declined after antimicrobial and supportive treatment. On day 6, abrupt biomarker re-elevation, high fever, progressive abdominal distension, and hemodynamic deterioration prompted repeat CT, which demonstrated extraluminal gas adjacent to the sigmoid colon. Emergency laparotomy confirmed complete sigmoid transection with partial stump necrosis and extensive fecal contamination. The patient survived after colectomy, colostomy, prolonged intensive care, and staged pelvic fixation. This case shows that bowel injury may continue to progress despite repeated negative CT and ultrasonographic examinations and transient improvement in inflammatory markers. When a high-risk mechanism and the clinical trajectory remain discordant, renewed surgical assessment and escalation beyond repeated noninvasive observation should be considered.

## 1 Introduction

High-energy pelvic ring injuries place a significant burden on trauma centers and often occur in conjunction with severe concomitant injuries (1). Colorectal injury is uncommon in this setting but may have serious clinical consequences (2). If bowel injury is missed or diagnosed late, fecal contamination may transform an initially closed pelvic fracture into a functionally open injury. Mortality after open pelvic ring injury has been reported to approach 50%, with late deaths commonly attributed to wound sepsis and multiple organ dysfunction (3, 4). Here, we describe an unusual case of delayed complete transection of the sigmoid colon following high-energy pelvic trauma. Complete transection is not a distinct clinical entity merely because it is anatomically more extensive than limited perforation, and both require timely source control once fecal contamination occurs. The value of the present case lies instead in its unusually severe and concealed diagnostic course. In a patient who could not be examined reliably, CT on post-injury days 1–3, FAST, and repeated ultrasonography remained non-diagnostic, while some laboratory indices temporarily improved. The bowel injury became apparent only on day 6 after abrupt clinical deterioration. This case illustrates the discordance that may arise between a high-risk injury mechanism, serial negative examinations, transient biochemical improvement, and continued progression of an uncontrolled structural source.

## 2 Case presentation

### 2.1 Clinical presentation

In June 12, 2025, a 57-year-old woman was brought to the emergency department of our hospital two hours after sustaining multiple pelvic fractures in a traffic accident. Upon presentation, the patient was in poor condition, suffering from hemorrhagic shock. At emergency admission, her temperature was 38.5 °C, pulse rate was 115 beats/min, respiratory rate was 28–32 breaths/min, and blood pressure was 97/68 mmHg. During the first 15 h, urine output was 700 mL; total infused volume was 3,539 mL, and gastric decompression output was 800 mL. On abdominal palpation, the abdomen was soft, with no tenderness or rebound tenderness; the abdominal contour and bowel sounds were normal. Additionally, a preliminary abdominal CT scan showed no evidence of significant bowel injury (Figure 1A). The patient had previously been in good health and had no relevant medical history. Emergency FAST demonstrated only a small amount of scattered abdominopelvic fluid. Due to severe pelvic ring injuries accompanied by hemorrhagic shock, the patient gradually slipped into a coma and was immediately transferred to the ICU for further treatment. The patient's detailed clinical course is shown in Table 1.

**Figure 1:**
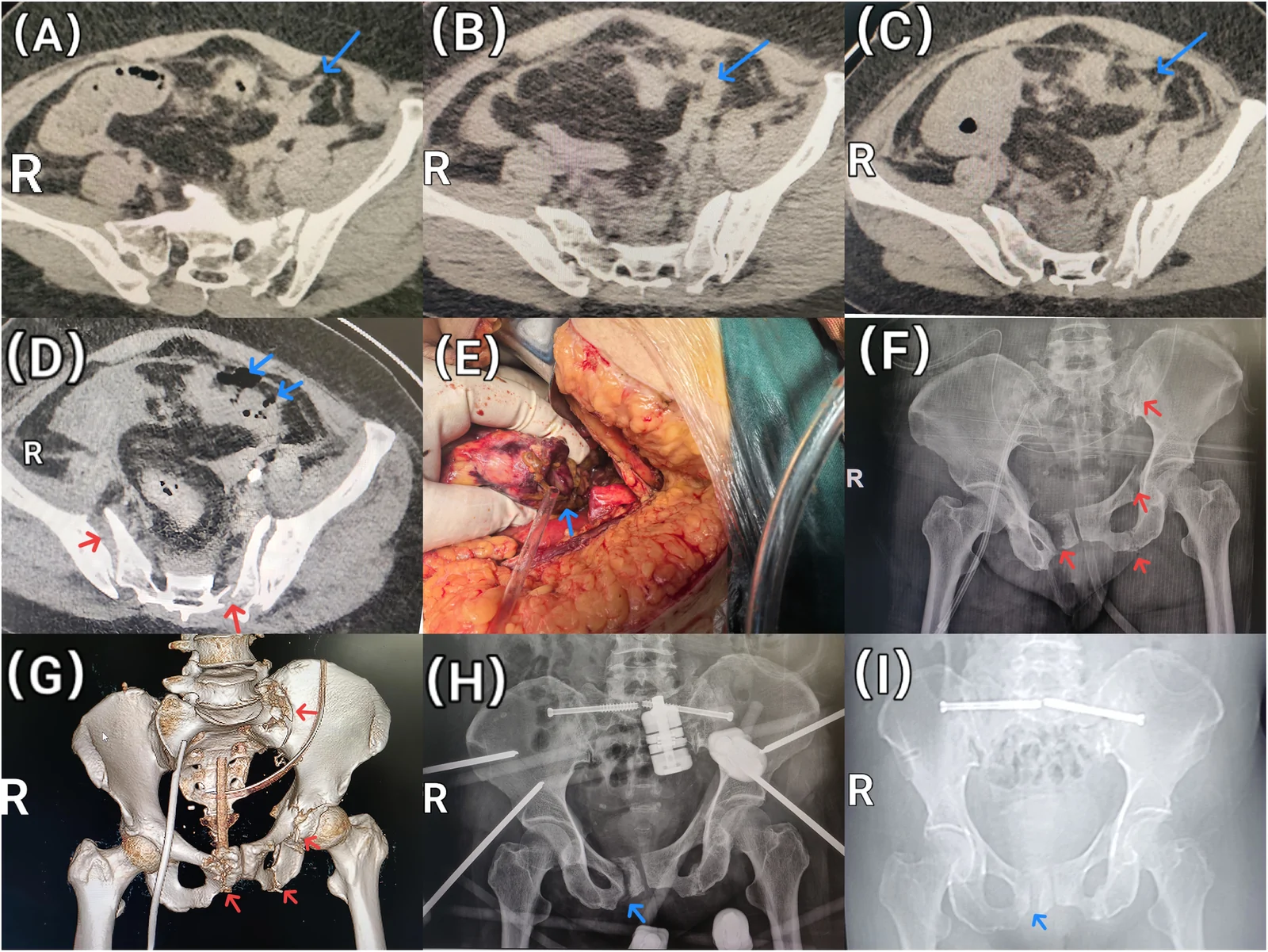
Imaging, intraoperative findings. **(A–C)** Representative images from serial abdominal CT scans obtained over 3 consecutive days, showing no evidence of bowel wall perforation or extraluminal gas (blue arrows). **(D)** Repeat CT demonstrated free intraperitoneal gas near the sigmoid colon (blue arrows), together with a left sacral fracture and right sacroiliac joint disruption (red arrows). **(E)** Intraoperative view showing complete transection of the sigmoid colon, active bleeding, partial stump necrosis, and extensive purulent contamination within the abdominopelvic cavity. **(F)** Initial pelvic radiograph and **(G)** three-dimensional CT reconstruction showing a Young–Burgess lateral compression type III pelvic ring injury, including fractures of the left sacrum, bilateral pubic rami, and left ischium, as well as a concomitant left acetabular fracture (red arrows). **(H)** Radiograph obtained 10 weeks after fracture fixation, showing localized bone resorption consistent with infection-related change (blue arrow). **(I)** Radiograph obtained at the 7-month follow-up after fracture fixation, demonstrating persistent localized bone resorption after removal of the anterior pelvic ring–acetabular external fixation construct (blue arrow).

**Table 1:** Clinical timeline.

Time point	Clinical findings and diagnostic reassessment	Major interventions and outcomes
Admission day (day of injury, June 12)	GCS 14; severe pelvic ring and acetabular fractures; hemorrhagic shock; soft, non-tender abdomen without rebound tenderness; emergency FAST showed small-volume abdominopelvic fluid; bowel sounds normal; respiratory rate 28–32/min; blood pressure 97/68 mmHg; during the preceding 15 h, the total fluid intake was 3,539 mL, whereas urine output was 700 mL; initial CT showed no direct signs of bowel injury;	ICU admission; respiratory and circulatory support; pelvic binder; skeletal traction; norepinephrine support; Early admission: Enteral nutrition withheld; gastrointestinal decompression.
Post-injury day 1	GCS 3; worsening physiology and early organ dysfunction; bowel sounds reduced (1/min); respiratory rate 38–42/min; blood pressure 95/61 mmHg; during the preceding 24 h, the total fluid intake was 6,179 mL, whereas urine output was 700 mL; June 13 CT showed no bowel dilatation, bowel-wall thickening, or free intraperitoneal gas	Endotracheal intubation on June 13; high-dose norepinephrine required to maintain blood pressure; continued close abdominal reassessment; blood products transfused; analgesia and sedation administered
Post-injury day 2	GCS 3; blood pressure 109/63 mmHg; bowel sounds reduced (1/min); repeat CT showed no bowel fluid accumulation, dilatation, or wall thickening	Hemodynamics improved; norepinephrine dose reduced
Post-injury day 3	Bowel sounds no longer audible; during the preceding 24 h, the total fluid intake was 2,918 mL, whereas urine output was 68 mL;	Diagnostic abdominal paracentesis performed after surgical reassessment; renal failure with oliguria; CRRT initiated; diagnostic abdominal paracentesis was performed, but no diagnostically useful intraperitoneal fluid was obtained; NRS-2002 score >3; parenteral nutrition was initiated, with gastrointestinal decompression continued.
Post-injury day 4–5	Bowel sounds remained absent; no definite peritoneal signs could be established because of impaired consciousness; anuria; improved inflammatory markers and hemodynamic status (blood pressure 120/60 mmHg)	Norepinephrine discontinued; continued organ support, antimicrobial therapy, and close clinical monitoring
Post-injury day 6 (June 18)	High fever, progressive abdominal distension, and worsening hemodynamic instability while norepinephrine support continued; inflammatory markers increased sharply; the patient's condition deteriorated, with a blood pressure of 88/54 mmHg; during the preceding 18 h, the total fluid intake was 5,753 mL, whereas urine output was only 31 mL; repeat CT showed free gas adjacent to sigmoid colon	High-dose norepinephrine required to maintain blood pressure; emergency laparotomy; complete sigmoid transection confirmed; colectomy
Hospital days 7–34	Gradual improvement in septic shock and multiorgan dysfunction; persistent pelvic drainage and local infection remained; June 30: Sedation and analgesia discontinued; consciousness recovered, with eye opening, nodding to commands, and brisk pupillary light reflexes	ICU care; postoperative day 1: small-volume glucose water administered via nasogastric tube for trophic tolerance assessment; postoperative day 2: peptide-based enteral nutrition initiated; parenteral nutrition continued during transition, followed by gradual advancement to a liquid oral diet; tracheostomy (July 3); CRRT was discontinued on July 5; broad-spectrum antibacterial and antifungal therapy; organ support; norepinephrine was gradually tapered and discontinued on July 1
Hospital day 34	Vital signs were stable	Definitive pelvic ring and acetabular fixation; postoperative ICU admission
Hospital day 97	Infection and organ dysfunction improved sufficiently for step-down care	Transfer from ICU to orthopedic ward
Hospital day 117	Persistent colostomy, tracheostomy, and lower-limb weakness; poor surgical wound healing with superficial local skin and soft-tissue infection	Discharge from orthopedic ward
7-month follow-up after fracture fixation	Improved proximal lower-limb strength; persistent distal weakness and localized pelvic infection	Ongoing rehabilitation and follow-up

Timeline of events, interventions, and findings.

### 2.2 Examinations and diagnosis

Emergency CT demonstrated a Young–Burgess lateral compression type III pelvic ring injury, characterized by fractures of the left sacrum, left ischium, and bilateral pubic rami, together with right sacroiliac joint disruption. A concomitant fracture of the left acetabulum was also identified (Figures 1F,G). The patient also had traumatic intracranial hemorrhage, acute respiratory failure, contusions of the liver, right kidney, and spleen, multiple rib and sternal fractures, a severe left-elbow soft-tissue contusion with an open wound, and a small hemoperitoneum. Laboratory tests showed a serum lactate concentration of 5.6 mmol/L, a procalcitonin (PCT) level of 4.29 ng/mL, and an interleukin-6 (IL-6) concentration of 2,348 pg/mL; meanwhile, the white blood cell count was 8.0 × 10^9^/L, and the high-sensitivity C-reactive protein (hsCRP) was within the normal range at 6.38 mg/L. Cefuroxime sodium was initiated empirically for pulmonary contusion with suspected concomitant pulmonary infection in the setting of elevated inflammatory markers. Comprehensive bedside ultrasonography after ICU admission showed no specific sign of bowel injury and no increase in the small-volume abdominopelvic fluid. However, on the second day after the injury, the patient's condition deteriorated significantly; a neurological examination revealed a Glasgow Coma Scale (GCS) score of 3. Within 24 h of admission to the ICU, the patient's APACHE II score was 41, with a predicted mortality rate of 92%. Bowel sounds became reduced (1/min). Repeat chest and abdominal CT scans of the patient showed no significant changes compared to the scans taken at admission (Figure 1B); there was no increase in intra-abdominal hemorrhage, and no signs of bowel injury were found. Laboratory testing showed a white blood cell count exceeding 20 × 10^9^/L, with neutrophils accounting for 89.5%. High-sensitivity C-reactive protein had risen to 186.3 mg/L, procalcitonin to 39.0 ng/mL, and interleukin-6 to more than 5,000 pg/mL. Arterial blood gas analysis was consistent with metabolic acidosis, showing a pH of 7.32, base excess of −7 mmol/L, and lactate elevation to 4.2 mmol/L. These abnormalities reflected worsening systemic inflammation and progressive organ dysfunction. Therefore, empirical treatment with meropenem was initiated. On June 13, tachypnea progressed to 40 breaths/min, and endotracheal intubation with mechanical ventilation was required. By post-injury day 3, bowel sounds were no longer audible. PCT, IL-6, and some hepatic and renal indices decreased from their early peaks. Although delayed intestinal injury was considered possible, serial abdominal CT scans over 3 consecutive days showed no evidence of bowel wall perforation or extraluminal gas (Figures 1A–C). The hemoperitoneum and retroperitoneal hematoma were considered likely related to the concomitant pelvic and acetabular fractures and liver, splenic, and renal injuries, with no significant increase in bleeding. Diagnostic abdominal paracentesis was performed within the first 3 days after admission, but only a minimal amount of intraperitoneal fluid was obtained, which was insufficient for diagnostic evaluation. Following aggressive resuscitation, although severe organ dysfunction persisted, including renal failure, the patient's hemodynamic status improved and inflammatory markers decreased. Therefore, exploratory laparotomy was not considered indicated at that time. The enteral nutrition was withheld from the day of injury (June 12) because of hemorrhagic shock and impaired gastrointestinal function, and continuous gastric decompression was instituted. On post-injury day 3, after the NRS-2002 score exceeded 3, parenteral nutrition with intravenous lipid emulsion, glucose, and amino acids was initiated; enteral nutrition remained withheld. On post-injury day 6, these markers rose abruptly again (Figures 2A,B), accompanied by high fever, progressive abdominal distension, and worsening hemodynamic instability. During the preceding 18 h, fluid intake totaled 5,753 mL whereas urine output was 31 mL. Urgent CT then demonstrated extraluminal gas near the sigmoid colon (Figure 1D). Emergency laparotomy confirmed complete sigmoid transection (Figure 1E).

**Figure 2:**
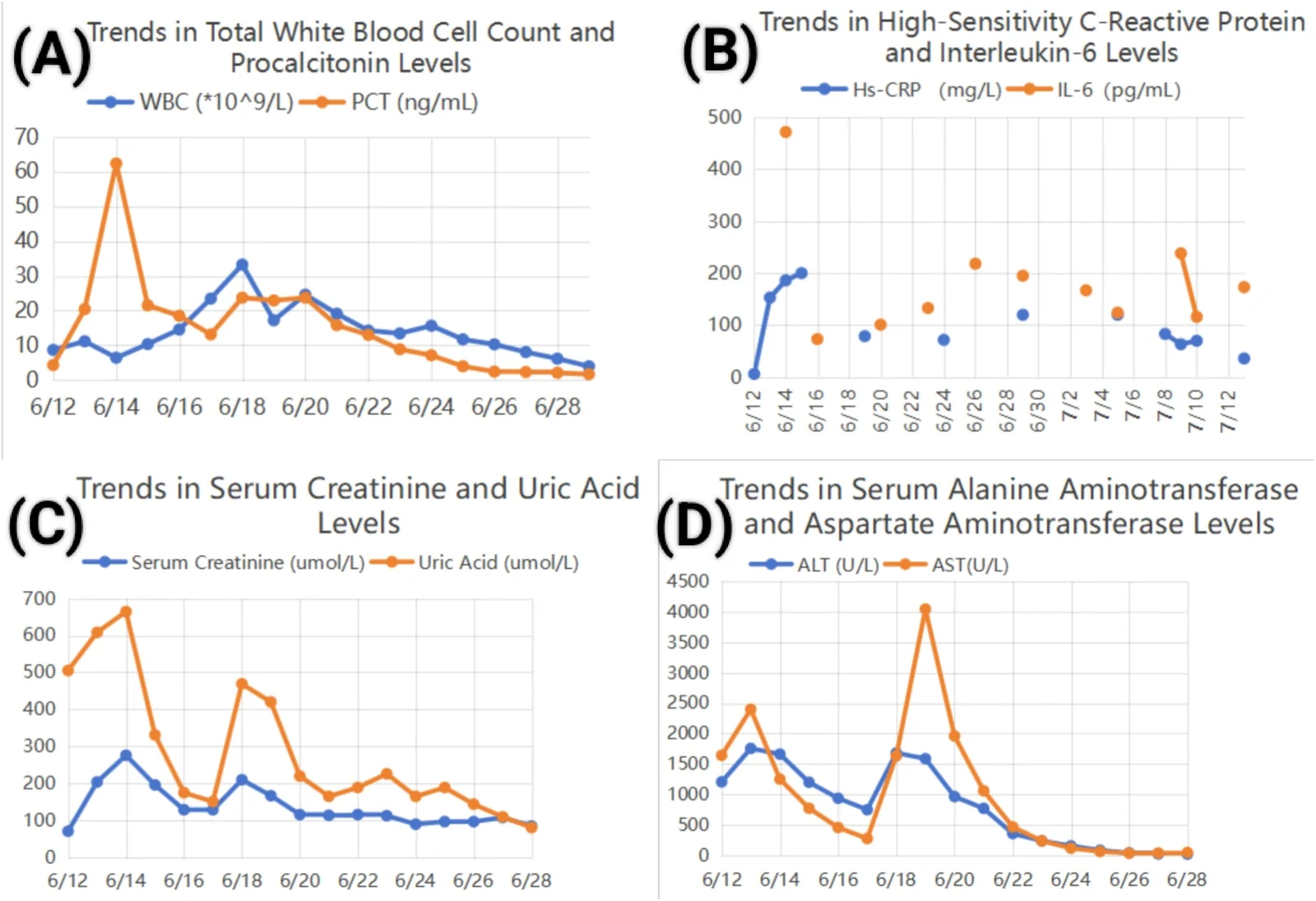
Laboratory trends during the clinical course. **(A,B)** PCT, IL-6, and other inflammatory markers increased markedly during the early post-injury period, partially declined after antimicrobial and supportive treatment, and subsequently increased again around post-injury day 6. **(C,D)** Hepatic and renal function indices deteriorated around the time of diagnosis, as reflected by increased creatinine, uric acid, alanine aminotransferase, and aspartate aminotransferase levels, followed by gradual improvement after emergency laparotomy.

### 2.3 Therapeutic intervention

Upon ICU admission, mechanical ventilation, restrictive fluid resuscitation, and blood transfusion were started to stabilize respiratory and hemodynamic function. In the initial stages of the damage control strategy, we performed closed reduction of the left hip dislocation, applied tibial skeletal traction to the left lower extremity, and used a pelvic fixation belt for temporary pelvic stabilization. Supportive care also included stress-ulcer prophylaxis and nebulized airway treatment. On post-injury day 3, the patient developed renal failure with anuria and was treated with continuous renal replacement therapy (CRRT); hepatic failure also developed, and hepatoprotective therapy was administered. After a repeat CT demonstrated free intraperitoneal gas on post-injury day 6 (Figure 1D), emergency exploratory laparotomy was performed. Intraoperatively, approximately 1,500 mL of mixed hemorrhagic and feculent intraperitoneal fluid was found, and complete transection of the sigmoid colon was confirmed, with active bleeding from the transected ends, partial stump necrosis, and ongoing fecal spillage (Figure 1E). Partial sigmoid colectomy, sigmoid colostomy, abdominal and pelvic debridement, drainage, and sigmoid suspension were performed to achieve source control and prevent postoperative volvulus or torsion. Given septic shock, extensive contamination, and nonviable bowel ends, primary anastomosis was not selected. Postoperatively, the patient was transferred to the ICU. The patient had severe multiple organ damage, and the estimated Injury Severity Score (ISS) was 66, indicating extremely severe trauma with a very low survival rate. On postoperative day 1, weak gastrointestinal motility permitted only small-volume glucose water through a nasogastric tube as a trophic tolerance assessment; amino-acid-based parenteral nutrition continued. On postoperative day 2, after no obvious gastric retention following water administration, peptide-based enteral nutrition was initiated through the nasogastric tube and combined with parenteral nutrition. Marked gastric retention on postoperative day 13 prompted temporary cessation of the enteral formula. Feeding was gradually advanced as gastrointestinal function recovered, and fruit juice and rice porridge were introduced approximately one month after colectomy. Vancomycin was added on June 18 to broaden Gram-positive coverage during septic deterioration. As the patient was in a state of septic shock and had risk factors for invasive fungal infection, the antimicrobial regimen was further broadened and adjusted to include vancomycin, imipenem-cilastatin, and caspofungin. Pelvic purulent-fluid and ascitic-fluid specimens collected on June 18 grew Enterococcus faecium. Caspofungin was changed to micafungin on June 21 because hepatic dysfunction worsened; vancomycin was dose-adjusted by therapeutic drug monitoring and antibacterial agents by estimated glomerular filtration rate. Later, microbiology included Candida glabrata in urine, and Stenotrophomonas maltophilia plus Enterococcus faecium in blood cultures. Respiratory cultures grew Stenotrophomonas maltophilia and Enterobacter cloacae. Despite repeated dressing changes, the severe left-elbow contusion wound continued to produce copious purulent drainage. Culture of the wound exudate grew Mucor circinelloides. Invasive mucormycosis was therefore suspected, and liposomal amphotericin B was initiated. Antimicrobial dosing was guided by serum drug concentrations, organ function, CRRT, and multidisciplinary consultation (Table 2). The patient's signs of sepsis gradually resolved, and hepatic and renal function improved (Figures 2C,D). CRRT was discontinued on July 5 after urine output improved, and furosemide was used to facilitate diuresis. By post-injury day 34, systemic sepsis and organ dysfunction had improved sufficiently for definitive pelvic fixation; this did not indicate eradication of all local infection. Infection had not been eradicated: gray purulent pelvic drainage persisted, and microbiological examination identified Proteus penneri and Escherichia coli. Culture-directed treatment was continued. Because local pelvic infection remained a concern, two cannulated screws and four Steinmann pins connected to external rods were used to form a hybrid internal–external construct, providing stability while limiting the amount of internal hardware (Figure 1H). Following definitive fixation of the pelvic ring and acetabular fractures, markers of systemic infection continued to decline. The antimicrobial regimen was subsequently adjusted over time to include levofloxacin, cefoperazone-sulbactam, and piperacillin-tazobactam. About 3 weeks later, this was changed to cefuroxime for maintenance therapy. The timeline of antimicrobial therapy during hospitalization is presented in Table 2.

**Table 2:** Antimicrobial therapy and documented microbiological timeline during hospitalization.

Period	Antimicrobial regimen	Dose and route
Admission to day 2	Cefuroxime	1.5 g IV every 8 h; empirical treatment for suspected early pulmonary infection
Day 2–Day 8	Meropenem	1 g IV every 12 h; rapid clinical deterioration with sharp increases in PCT, WBC, and hs-CRP
Day 6–Day 15	Vancomycin	0.5 g IV every 8 h; broadened Gram-positive coverage due to septic deterioration; pelvic purulent-fluid and ascitic-fluid cultures collected June 18 grew Enterococcus faecium
Day 8–Day 10	Caspofungin	35 mg IV once daily; sepsis with high risk of fungal infection
Day 10–Day 19	Micafungin	100 mg IV once daily; changed from caspofungin because hepatic dysfunction worsened; discontinued after urine culture on June 28 grew Candida glabrata >10^4^ CFU/mL and fluconazole was initiated for suspected fungal urinary tract infection
Day 15–Day 25	Vancomycin	0.5 g IV every 12 h; continued vancomycin
Day 19–Day 26	Fluconazole	400 mg IV qd; initial loading dose 800 mg
Day 21–Day 25	Imipenem-cilastatin	1 g IV every 6 h; broadened antimicrobial coverage for severe septic shock
Day 25–Day 30	Vancomycin	0.5 g IV once daily; continued vancomycin
Day 25–Day 29	Imipenem-cilastatin	1 g IV every 12 h; continued imipenem-cilastatin
Day 26–Day 46	Liposomal amphotericin B	300 mg IV once daily; initiated after purulent drainage culture grew Mucor circinelloides in the setting of a persistently purulent left-elbow wound and suspected invasive mucormycosis
Day 29–Day 47	Levofloxacin	0.5 g IV once daily; used from July 11 for sepsis and bacterial pneumonia; blood cultures grew Stenotrophomonas maltophilia and Enterococcus faecium, while respiratory cultures grew Stenotrophomonas maltophilia and Enterobacter cloacae; trimethoprim-sulfamethoxazole was later added because fever persisted
Day 29–Day 37	Trimethoprim-sulfamethoxazole	160/800 mg via nasogastric tube every 8 h
Day 29–Day 32	Cefoperazone-sulbactam	3 g IV every 8 h; empirical combination therapy for bacterial pneumonia from July 11; subsequent treatment was adjusted according to clinical response and organ function
Day 35–Day 53	Piperacillin-tazobactam	4.5 g IV every 8 h; used in the later infection-control phase
Day 53–Day 56	Cefuroxime	1.5 g IV every 8 h; used in the later infection-control phase

IV, intravenous. Antimicrobial dosing was guided by serum drug concentrations, organ function, CRRT, and multidisciplinary consultation.

### 2.4 Follow-up and outcomes

Multidisciplinary management contributed to the patient's survival despite a prolonged and complicated clinical course. Following a 97-day ICU stay, the patient's clinical condition had improved sufficiently for transfer to the orthopedic ward. During the subsequent 20-day stay on the orthopedic ward, the anterior pelvic ring–acetabular external fixation construct was removed, after which she was discharged. At discharge, significant infection-related sequelae remained, including severe bilateral lower-extremity weakness (left lower limb, MRC grade 1; right lower limb: proximal muscles, MRC grade 1, and distal muscles, MRC grade 0), consistent with critical illness polyneuropathy/myopathy secondary to sepsis and prolonged critical illness. Additional complications included localized infection-related bone resorption at the pelvic fracture site (Figure 1E), a persistent sigmoid colostomy, and a retained tracheostomy tube. At the 7-month follow-up after pelvic ring and acetabular fracture fixation, muscle strength proximal to the knees had improved to MRC grade 3 in both lower limbs, whereas distal strength had improved to MRC grade 1. A localized pelvic infectious focus persisted (Figure 1I).

## 3 Discussion

Several diagnostic challenges were evident in retrospect. Early abdominal assessment may have been confounded by several factors, including the presence of a retroperitoneal hematoma, and impaired responsiveness related to the concomitant head injury, all of which could have reduced the sensitivity of abdominal examination for detecting peritonitis (2). Initial CT may also be falsely reassuring; once free gas becomes visible, full-thickness bowel necrosis may already have occurred (5, 6). The early elevation of inflammatory markers was initially considered in the differential diagnosis to possibly reflect pulmonary or urinary tract infection. However, early sputum, blood, and urine cultures showed no bacterial growth after 5 days. All positive microbiological findings were obtained after sigmoid colon transection had been diagnosed at laparotomy on June 18. In retrospect, the available evidence did not support pulmonary or urinary tract infection as the explanation for the early inflammatory abnormalities. Bowel sounds were normal on admission, became reduced on post-injury day 2, and were absent from post-injury day 3 onward. Following repeat surgical assessment on post-injury day 3, diagnostic abdominal paracentesis was performed, but no diagnostically useful intraperitoneal fluid was obtained. The general surgery team considered that the dependent pelvic location and partial containment of the hemoperitoneum may have limited the diagnostic yield. However, in patients with high-energy pelvic trauma, particularly when clinical assessment is limited, persistent or increasing suspicion should prompt close observation, serial clinical and laboratory reassessment, and consideration of repeat contrast-enhanced CT. Surgical exploration may be warranted if the patient deteriorates or significant bowel injury is suspected despite negative or equivocal imaging findings.

To our knowledge, very few case reports have described sigmoid colon injury associated with pelvic fracture. Santana-Cabrera et al. reported a delayed sigmoid colon perforation 5 days after a major pelvic fracture caused by a motor vehicle collision, with no bowel injury detected on initial CT (7). Shapiro and Wolverson described retroperitoneal sigmoid colon perforation secondary to fracture-dislocation of the sacroiliac joint (8). More recently, delayed bowel injury associated with pelvic fracture has also been reported in other unusual clinical contexts (9). In the present case, the delayed bowel injury was confirmed intraoperatively as complete transection of the sigmoid colon, rather than a limited perforation or bowel-wall laceration. We agree that complete transection is not a separate clinical entity simply because it is more extensive than limited perforation. Both can cause fecal contamination and sepsis and require prompt source control. The present case is clinically distinctive because a severe injury evolved under unusually concealed conditions despite contemporary serial CT, FAST, bedside ultrasonography, and intensive monitoring. It became apparent only after progression to complete loss of bowel continuity, stump necrosis, extensive fecal contamination, septic shock, and multiorgan dysfunction. The clinical contribution of this case therefore lies not in the anatomical term “complete transection” alone, but in the mismatch between repeated negative CT and ultrasonographic findings, temporary improvement in inflammatory markers, and the continued progression of the bowel injury. Following high-energy pelvic trauma, delayed ischemic bowel injury should be considered even when the initial abdominal examination reveals no abnormalities.

Following high-energy pelvic trauma, even if an initial abdominal examination reveals no abnormalities, delayed ischemic bowel injury should be considered. In this case, the fracture site may have exerted traction or shearing forces on the mesenteric vessels, leading to bowel-wall ischemia, which in turn caused delayed necrosis and perforation (5). In addition, we considered that, following high-energy pelvic trauma, mesenteric compression by hematoma together with severe hemorrhagic shock may have aggravated intestinal hypoperfusion and microcirculatory dysfunction, leading to ischemic injury and ultimately contributing to delayed bowel perforation. No evidence of direct penetration or impingement was identified during the surgical procedure. Partial necrosis of the transected ends was compatible with progressive ischemia after mesenteric traction or shearing. Because no angiographic or targeted histopathological evidence demonstrated mesenteric vascular disruption, an initial direct mechanical component cannot be excluded.

Notably, the laboratory and imaging course also requires cautious interpretation. PCT, IL-6, and some organ-function indices partially declined after their early peaks; these changes were supportive rather than decisive and did not demonstrate source control. The June 18 CT was prompted by high fever, progressive abdominal distension, hemodynamic deterioration, and renewed laboratory abnormality, rather than by a single biomarker value. Similarly, FAST and bedside ultrasonography were useful for monitoring fluid and associated injuries but could not exclude bowel or mesenteric injury. The World Society of Emergency Surgery guidelines emphasize that a negative or equivocal initial CT does not exclude bowel or mesenteric injury in a high-risk patient. Close observation, serial clinical and laboratory assessment, and repeat CT are appropriate when suspicion persists; surgical exploration should be considered when the patient deteriorates or clinical findings indicate significant bowel injury (5). The Bowel Injury Prediction Score (BIPS) assigns one point each for abdominal tenderness, a white blood cell count of at least 17,000/mm^3^, and CT evidence of significant mesenteric injury. A score of 2 or more is associated with an increased risk of blunt bowel injury requiring operative management (10). In this case, the utility of BIPS was limited because abdominal tenderness could not be reliably assessed and early serial CT examinations showed no significant mesenteric injury. Although the patient's overall condition improved following resuscitation, with decreasing inflammatory markers and stabilization of hemodynamics, marked leukocytosis and the progressive loss of bowel sounds remained concerning, highlighting the need for continued observation and serial clinical reassessment.

This case supports a practical escalation principle: In a clinically non-evaluable patient, serial negative imaging indicates only that specific signs are absent at the time of examination. When a high-risk mechanism, evolving gastrointestinal findings, and the systemic trajectory remain inconsistent with those results, clinicians should reconsider the working diagnosis, obtain renewed surgical review, and change the diagnostic approach. Depending on physiological stability, options include diagnostic peritoneal sampling, diagnostic laparoscopy, or exploratory laparotomy rather than repeated observation or antimicrobial escalation alone.

Several limitations remain: this single retrospective case cannot establish an LC-III-specific mechanism; the timing and vascular basis of the transection were not directly demonstrated.

## 4 Conclusion

The clinical value of this case does not lie in complete sigmoid transection as an anatomical distinction alone. It shows that severe structural bowel injury may progress despite serial negative CT, FAST, bedside ultrasonography, and transient biochemical improvement in a clinically non-evaluable patient. Persistent discordance between a high-risk injury mechanism and the clinical course should prompt renewed surgical assessment and, when noninvasive evaluation remains inconclusive, consideration of invasive diagnosis or operative exploration. Repeated clinical reassessment and timely control of the source of infection can reduce the risk of further deterioration.

## 5 Patient perspective

The patient and her family expressed their gratitude for the care they received during the hospital stay. They particularly acknowledged the contribution of the multidisciplinary team, including the surgical, intensive care, and nursing teams, in managing her complex condition.

## Data Availability

The raw data supporting the conclusions of this article will be made available by the authors, without undue reservation.
